# Breakdown of Inter-Hemispheric Connectivity Is Associated with Posttraumatic Symptomatology and Memory Impairment

**DOI:** 10.1371/journal.pone.0144766

**Published:** 2016-02-10

**Authors:** Rotem Saar-Ashkenazy, Ronel Veksler, Jonathan Guez, Yael Jacob, Ilan Shelef, Hadar Shalev, Alon Friedman, Jonathan E. Cohen

**Affiliations:** 1 Department of Brain and Cognitive Neuroscience, The Zlotowski center for Neuroscience Ben-Gurion University of the Negev, Beer-Sheva, Israel; 2 Department of Psychology and the School of Social-work, Ashkelon Academic College, Ashkelon, Israel; 3 Department of Physiology and Cell Biology, Ben-Gurion University of the Negev, Beer-Sheva, Israel; 4 Department of Psychology, Achva Academic College, Beer-Tuvia regional council, Israel; 5 Beer-Sheva Mental Health Center, Beer-Sheva, Israel; 6 Sagol School of Neuroscience and The Functional Brain Imaging Unit, Wohl Institute for Advanced Imaging, Tel Aviv University, Israel; 7 Department of Radiology, Soroka University Medical Center, Beer-Sheva, Israel; 8 Department of Psychiatry, Soroka University Medical Center, Beer-Sheva, Israel; 9 Department of Medical Neuroscience, Faculty of Medicine, Dalhousie University, Halifax, Nova Scotia, Canada; 10 Sharett Institute of Oncology, Hadassah Hebrew University Medical Center, Jerusalem, Israel; Central Institute of Mental Health, GERMANY

## Abstract

Altered brain anatomy in specific gray-matter regions has been shown in patients with posttraumatic stress disorder (PTSD). Recently, white-matter tracts have become a focus of research in PTSD. The corpus callosum (CC) is the principal white-matter fiber bundle, crucial in relaying sensory, motor and cognitive information between hemispheres. Alterations in CC fibers have been reported in PTSD and might be assumed to underlie substantial behavioral and cognitive sequelae; however most diffusion tensor imaging (DTI) studies in adult-onset PTSD failed to address the clinical correlates between imaging and PTSD symptoms severity, behavioral manifestation and cognitive functions. In the current study we examined (a) to what extent microstructural integrity of the CC is associated with memory performance and (b) whether imaging and cognitive parameters are associated with PTSD symptom severity. DTI data were obtained and fractional anisotropy (FA) values were computed for 16 patients and 14 controls. PTSD symptom severity was assessed by employing the clinician administered PTSD scale (CAPS) and memory was tested using a task probing item and associative memory for words and pictures. Significant correlations were found between PTSD symptoms severity, memory accuracy and reaction-time to CC FA values in the PTSD group. This study demonstrates meaningful clinical and cognitive correlates of microstructural connectivity. These results have implications for diagnostic tools and future studies aimed at identifying individuals at risk for PTSD.

## Introduction

Post-traumatic stress disorder (PTSD) is a debilitating condition that can develop in response to an acute traumatic event and is characterized by re-experiencing, avoidance, negative alterations in cognition and mood and hyper-arousal [1]. Neuroimaging studies in PTSD have revealed structural differences in specific gray-matter regions (e.g. anterior cingulate cortex, hippocampus and the amygdala), with smaller cortical volumes frequently reported in post-traumatic patients [2–4]. Recently, white-matter tracts have become a focus of research in PTSD patients, with white-matter volume decrease being more often reported than increase (reviewed in [5]).

The majority of studies testing the exact structural and functional topography of white-matter in PTSD used brain-magnetic resonance imaging (bMRI) that allowed volumetric/area measurements. Several studies in PTSD have shown a reduction in the volume and/or area of the corpus callosum (CC), with or without generalized white-matter atrophy [6–11]. While many of these studies were based on volumetric measuring of white-matter, recently, the focus has been shifted to studying the integrity of white-matter tracts. Diffusion tensor imaging (DTI) allows studying the role of structural brain connectivity, and provides a detailed assessment of fiber tracts by analyzing the restricted diffusion of water molecules and has increasingly been utilized to detect changes in structural connectivity of white-matter tracts in psychiatric populations. A common parameter obtained from DTI measurements is fractional anisotropy (FA), which is a quantitative indicator of white matter integrity, reflecting fiber density, axonal diameter, and myelination [12–16].

The corpus callosum (CC) is the principal white-matter fiber bundle connecting neocortical areas and plays an integral role in relaying sensory, motor and cognitive information [17]. The CC was reported to be involved in inhibitory performance (i.e., cognitive control; [18], processing speed and motor functions [19], executive functions and verbal learning [20], working and verbal memory [21] and high intellectual capabilities [22]). These findings support the hypothesis that efficient information transfer between hemispheres is crucial for high cognitive functions. Alterations in the architecture of CC fibers, as reported in PTSD patients, are thus expected to have substantial behavioral and cognitive sequelae depending on the exact location of injury/fiber loss.

To date, DTI studies in adult PTSD patients were limited to childhood trauma [23] or to children and adolescents with acute and/or focal trauma, e.g. traumatic brain injury (TBI) [24–28], with most studies reporting decreased FA values in various white-matter regions (e.g., left uncinate fasciculus, medial and posterior parts of the CC and the anterior cingulate). In contrast, increased FA values were reported in the anterior [29] and dorsal [30] cingulum and the left superior frontal gyrus [31]. While these studies show promise for further characterization of PTSD-related white-matter abnormalities (for review, see also [32], many of them critically lack supporting evidence for clinical correlates between PTSD symptoms severity, behavioral manifestation or cognitive functions and CC imaging measurements.

In the current study we aimed to examine (a) to what extent predefined imaging (FA of the CC) and cognitive measurements (memory accuracy and response time) are associated in PTSD, and (b) whether imaging and cognitive parameters are associated with PTSD symptom severity, in a group of adult-onset PTSD patients without TBI. Specifically and based on previous literature, we hypothesized that high symptom severity is associated with lower CC micro-structural integrity as well as with impaired memory performance.

## Materials and Methods

### Participants

Participants in the current study were 16 PTSD patients (10 males and 6 females) recruited from the trauma-center at Soroka University Medical Center (demographic and characteristics of PTSD subjects are presented in Table 1), and 14 control subjects (10 males and 4 females) that were recruited from the community (all reported intact everyday functioning, and no specific cognitive or other disabilities). No significant differences in age (M = 37 (years), SD = 12.447, M = 31.142 (years), SD = 9.542, for PTSD and controls, respectively, z = -0.733, p = .463) or education (M = 13.25 (years), SD = 1.732, M = 13.500 (years), SD = 1.870, for PTSD and controls, respectively, z = 1.466, p = .142) were found between groups. Exclusion criteria included participants under the age of 18 years, past TBI and/or psychiatric/neurological disorders, alcohol abuse, or the use of illicit drugs affecting the central nervous system. Participants with a medical contraindication to MRI examination were excluded from the study.

**Table 1:** Demographics and Characteristics of PTSD Subjects.

Subject	Sex	Age	Education (years)	Trauma	Medications	Bodily Physical Injury	Head Physical Injury
1	M	37	12	Military related	None	No	No
2	M	24	12	Fall from height	Sertraline 50mg/d	No	No
3	M	26	15	Military related	Sertraline 100mg/d	Yes	No
4	F	49	15	MVA	Escitalopram 10mg/d, Clonazepam 0.5m/d	No	Whiplash
5	M	25	12	MVA	None	Yes	No
6	M	37	12	Assault	Paroxetine 20mg/d	No	No
7	F	52	15	MVA	Venlafaxine 150mg/d	No	No
8	M	26	12	Work related	None	Yes	No
9	F	39	15	MVA + Terror attack	Fluoxetine 20mg/d	No	No
10	F	55	12	MVA	None	No	No
11	M	44	12	MVA	None	Yes	No
12	F	58	12	MVA	Paroxetine 20mg/d	No	No
13	F	24	12	MVA	Paroxetine 20mg/d	Yes	No
14	M	19	12	MVA	None	Yes	No
15	M	45	15	Fall from height	Sertraline 100mg/d	Yes	No
16	M	32	17	MVA	None	Yes	No

*Note*. Data is reported for all PTSD patients who participated in the current study; MVA = Motor Vehicle Accident.

All PTSD patients had experienced a traumatic event 6–36 months preceding the study. PTSD was diagnosed during a structured psychiatric interview using DSM-IV criteria performed by a board-certified psychiatrist (current co-morbid disorders were exclusionary). PTSD symptoms severity was assessed using the Clinician-Administered PTSD Scale (CAPS, [33]) which is a semi-structured interview that is designed to assess the essential features of PTSD and is widely considered to be the "gold standard" in PTSD assessment. CAPS scoring was performed according to the “1, 2” rule, which is used to determine a diagnosis; that is, a frequency score of 1 (scale 0 = "none of the time" to 4 = "most or all of the time") and an intensity score of 2 (scale 0 = "none" to 4 = "extreme") were required for a particular symptom to meet the criterion [34]. All procedures were approved by the Soroka-University Medical Center Institutional Review Board and all participants gave their written informed consent for participation (participants that were suspected to have compromised capacity/ability to consent were not included in the current study).

### Self-report Questionnaires

To assess for anxiety levels in both groups we used the Spielberger's State-Trait Anxiety Inventory (STAI); a self-report questionnaire that measures state and trait anxiety [35]. The total scores of this measure are obtained by summing the values assigned to each item and range from a minimum of 20 to a maximum of 80, with higher scores indicating more severe anxiety symptoms. Additionally, we used the Patient Distress Scale (PDS) questionnaire [36], with symptom severity score ranging from 0 to 51. Participants were given standardized instructions prior to completing the questionnaires.

### Memory task

**Fig 1:**
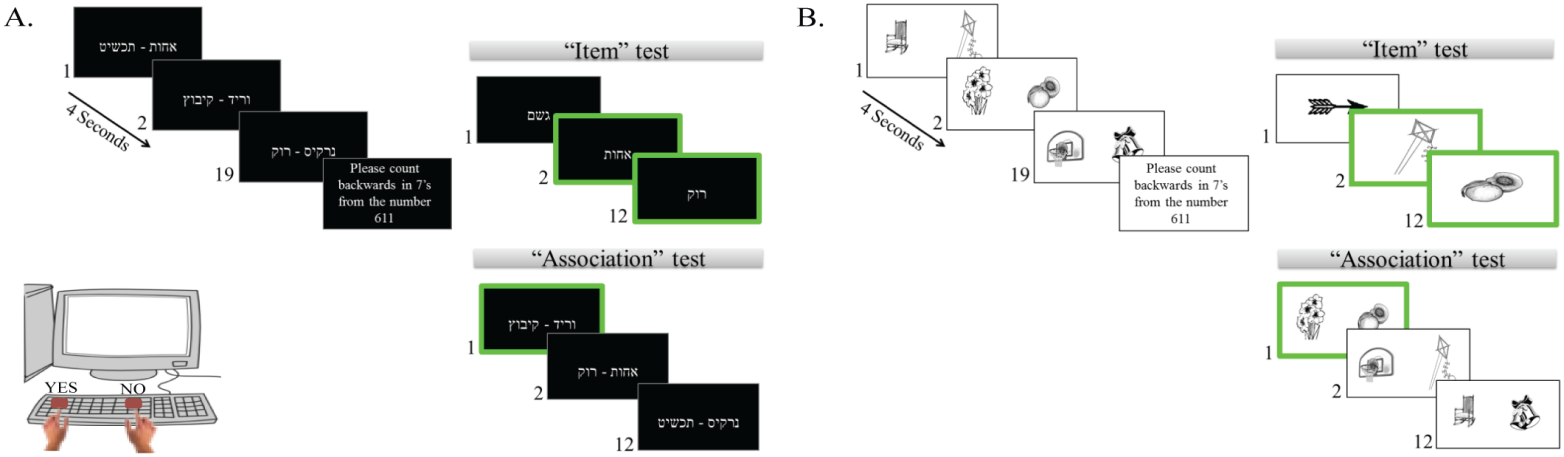
Experimental paradigm. Participants performed two types of memory tasks (A. words, B. pictures) with a similar construct: a learning phase that was followed by two repetitions for items and associative memory recognition. Participants were presented with a study list of emotionally neutral pairs. In the item recognition task, participants had to identify the 6 items that appeared in the study list and reject the others. In the associative recognition task, participants had to identify the 6 correct pairs, which appeared in the study list and reject the new, recombined pairs. Highlighted green rectangles indicate targets.

To probe for memory performance, we employed a previously used item-association memory paradigm (see [9, 37–38]) that included two types of memory tasks (words and pictures) with a similar construct (see Fig 1). All participants were given standardized instructions prior to the beginning of the memory paradigm. Participants performed one block of training (that included a learning list, followed by items and association recognition tests), followed by two experimental blocks for each task (words and pictures) that shared a similar structure (i.e. a learning list and one item and one association test). In the learning phase, participants were asked to study a list of 19 pairs of unrelated emotionally-neutral items, comprising 38 words/line-draw pictures, presented on a computer monitor, at a rate of 4 seconds per pair, randomized across participants. Stimuli were compiled from high-frequency common Hebrew nouns of unrelated (visually, semantically, or rhythmically) objects [39]. Learning was intentional: participants were instructed to learn both the individual stimuli and the pairs (different stimuli were used in each learning phase and list). The learning phase was followed by a 30-second distraction task (counting backward in sevens from a randomly selected number) to prevent rehearsal between the learning phase and memory task.

Participants performed the items recognition task, followed by the associative recognition task. In the items task, participants viewed 12 stimuli, one at a time. Of these, 6 were targets (i.e., original stimuli that had appeared during the learning phase), and 6 were distracters (i.e., new stimuli that had not appeared in the learning phase, but shared similar characteristics as the target stimuli), mixed randomly. Participants were informed that the list included targets and distracters, and were instructed to respond to each stimulus with a designated “yes” key (“1”) for targets and a “no” response key (“0”) for distracters. In the associative recognition task, participants viewed 12 stimuli-pairs; of those 6 were intact pairs from the learning phase (i.e., the same pairs that appeared in the learning phase) and the other 6 pairs were distracters (i.e., rearranged pairs that contained the same items from the learning list that were now recombined and presented as novel pairs). Participants were informed that the list included intact and recombined pairs, and were again instructed to respond using the same keys. Stimuli that were used in the items test were not used in the associative test and vice-versa. Response-time (RT, milliseconds) was recorded.

To assess performance, for each experimental block (i.e., two blocks of words and two blocks of pictures) we computed two outcome measures: for items performance and for associative performance, both calculated by the difference between the proportions of hits (responding "yes" to a target that had appeared in learning list of that specific block) minus the proportion of false-alarms (responding "yes" to a distracter that had not appeared in learning list of that specific block). This resultant in two items and two association performance measurements for each task (words and pictures). For analysis purposes, we averaged each measure (items and associative performance) across each task's blocks (words and pictures), thus remained with one items and one associative averaged performance measurements, for the words and pictures tasks separately. Subsequently, we created an associative deficit index (ADI) reflecting the difference between the averaged item recognition and the averaged associative recognition performance separately for each task (words, pictures). The ADI was calculated as the averaged items recognition proportion minus the averaged associative recognition proportion; thus, higher ADI score reflect greater associative deficit.

### Imaging data acquisition

#### MRI data acquisition

Structural MRI scans were acquired on a 1.5-Tesla scanner (Intera, Philips Medical Systems, Best), using a 6-channel SENSE head-coil. The pulse sequence used was a TIw-FFE (spoiled gradient echo). A T1-weighted whole brain anatomical scan was collected for each subject using the following parameters: repetition-time = 15ms, echo-time = 4.6ms, flip-angle = 30°, matrix-size 256x256, field of view 25.6 cm, 150 sagittal slices (1×1×1 mm resolution).

#### DTI data acquisition

DTI data were acquired on the same scanner using a single-shot echo-planar imaging (EPI) sequence with SENSE parallel imaging (reduction factor = 2.5). Axial slices of 3.0 mm thickness were acquired parallel to the anterior-posterior commissure line (AC-PC). The in-plane acquisition resolution was 2.88 × 3.58 mm. 42 slices were acquired with zero gap to cover the entire hemisphere and brainstem. The DTI data were acquired along 16 directions with b = 1000 s/mm^2^. A TR/TE = 5711/95 ms was used without cardiac gating for a total acquisition time of 3:08 min for each dataset.

### DTI Data Preprocessing & Fiber Tractography

All fiber tracking was performed using DtiStudio (H. Jiang, S. Mori; Johns Hopkins University, cmrm.med.jhmi.edu). At the first stage, we performed automatic image registration, i.e. registered the diffusion-weighted images to the b0-image (*b*-value = 1000) by applying affine linear registration [40], in order to correct distortions induced by eddy currents. Following this step, we applied the tensor calculation, which generated the apparent diffusion coefficient (ADC) map; this approach is based on the hypothesis that the size of the mid-sagittal area of the CC is related to the total number of fibers and, therefore, is an indicator of neural connectivity between the hemispheres [41]. CC shapes were outlined upon the 0-color map at the mid-sagittal level and binary 2D images were generated. Segmentation of the CC (performed using an in-house written Matlab script) corresponds to the scheme proposed by Witelson [42] that defines five vertical callosal segments based on specific arithmetic fractions of the maximum anterior–posterior extent. In particular, the CC is subdivided into regions comprising the anterior third, the anterior and posterior midbody, the posterior third, and the posterior one-fifth. For each segment, fiber tracking was performed with the following parameters: FA threshold 0.25 and an inner product threshold of 0.7, which prohibited angles larger than 70° during tracking. This step yielded at an average FA value for each CC portion, for each subject.

### Statistical Analysis

Mann-Whitney U-tests were used for between-group analysis. Pearson-correlations were employed to assess the strength of the linear correlation between imaging results and memory performance as well as symptoms severity. Analysis was performed using Statistica 9.0 software by StatSoft.

## Results

**Fig 2:**
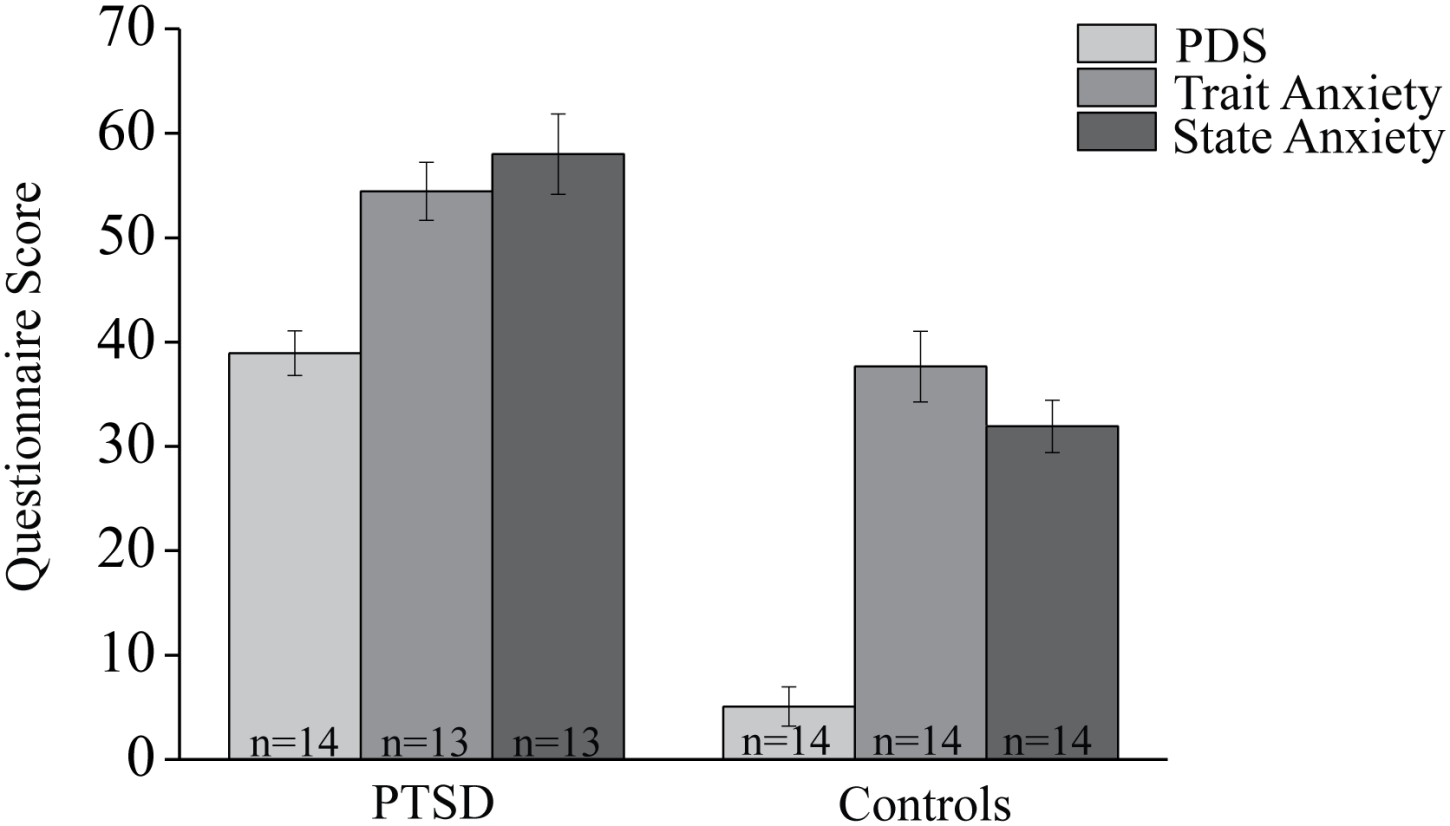
Questionnaire results. Significant differences between groups were found in the total PDS as well as the trait and state anxiety scores (p < .000 for all independent between-groups comparisons).

All analyses performed in the PTSD group were compared to an age and education matched control group. To verify the difference in anxiety levels between groups, trauma (PDS) and state-trait anxiety parameters were compared, and found significantly different with higher scores for the PTSD patients group (p < .001 for all independent between-groups comparisons, see Fig 2). Descriptive statistics of memory performance and imaging results for both groups (PTSD, control) are presented in Table 2. No significant differences were found between groups in memory performance or FA measurements.

**Table 2:** Descriptive Statistics.

		Min. Value PTSD	Min Value Controls	Max. Value PTSD	Max Value Controls	Mean Value PTSD	Mean Value Controls	Std. Dev PTSD.	Std. Dev. Controls
Memory Performance	ADI Words	-.667	-0.333	1.083	0.667	.242	0.348	0.492	0.297
	ADI Pictures	-.167	0.000	0.750	0.917	.235	0.492	0.278	0.356
	RT Item Words	799.083	823.083	2184.500	1553.125	1292.846	1107.879	430.479	236.573
	RT Association Words	967.458	1235.125	4052.500	1828.125	1732.646	1495.682	865.019	243.808
	RT Item Pictures	844.292	815.042	1414.000	1383.292	1144.313	1032.056	177.271	190.567
	RT Association Pictures	1144.534	1204.641	1700.500	1771.875	1435.595	1453.271	172.770	181.485
Fractional Anisotropy (FA)	Anterior CC	.492	.497	.583	.569	.541	.548	.028	.021
	Mid-Anterior CC	.489	.445	.598	.583	.531	.541	.032	.035
	Central CC	.518	.491	.612	.598	.561	.565	.027	.028
	Mid-Posterior CC	.513	.515	.601	.604	.561	.567	.023	.023
	Posterior CC	.509	.521	.646	.630	.583	.581	.034	.036
	Total CC	.526	.518	.519	.581	.555	.560	.021	.021

*Note*. ADI = associative deficit index; CON. = control subjects; RT = Reaction-time (milliseconds); FA = Fractional-anisotropy; CC = Corpus-Callosum; n PTSD = 16, n controls = 14.

To answer the question to what extent predefined imaging and cognitive measurements are associated in PTSD we computed the correlation between FA of the CC and ADI and RT in each group (PTSD/control) separately. Correlations are reported for the patients group only since no significant correlations were found in controls. To ensure that our results in the PTSD group are not influenced by participant's age or education we computed the correlation between these variables and cognitive and imaging parameters. Since the age of patients was found highly correlated to RT in the associative pictorial memory task (r = .695, p = .017) and the FA values of the anterior (r = -.627, p = .009) and the posterior (r = -.551, p = .027) CC portions, correlations reported below involving these variables were controlled for age.

Table 3 presents Pearson correlation coefficients between CC FA values, ADI and RT (one subject was excluded from the words task because Hebrew was not his native language). A significant correlation was found between the ADI in the word task and the total CC FA value (r = -.645, p = .044). To test whether this effect is specific to distinctive CC portions, we computed the correlation between the ADI in the word task and CC sub portions FA values and found that the source for this correlation is driven from the mid-posterior (r = -.641, p = .046) and posterior (r = -.679, p = .044) portions (uncorrected). No significant correlations were found with the ADI score on the pictorial task. RT in the associative pictorial task was highly correlated to the total CC FA value (r = -.648, p = .043), and the source for this effect was located to the anterior (r = -.657, p = .039) and central (r = -.667, p = .035) portions (uncorrected). In contrast, RT for items in both the word and the picture tasks, as well as RT for the associative words performance, was not correlated to CC FA values.

**Table 3:** Pearson Correlation Coefficients between Memory Performance Results and Corpus-Callosum Sub-Portion FA Values.

		CC FA Anterior^a^	CC FA Mid-Anterior	CC FA Central	CC FA Mid-Posterior	CC FA Posterior^a^	CC FA Total
Associative Deficit Index (ADI)	Words	-.599	.226	-.542	-.641*	-.679*	-.645*
	Pictures	.265	.196	.202	.464	.018	.186
Reaction Time(RT)	Mean Item RT (words)	.172	-.102	.239	.322	.543	.245
	Mean Association RT (words)	.183	-.290	-.089	.263	.393	.086
	Mean Item RT (pictures)	-.049	-.406	.063	.018	.121	.008
	Mean Association RT (pictures) ^a^	-.657*	-.248	-.667*	-.565	-.491	-.648*

*Note*. ADI = associative deficit index; CC sub-portions = corpus-callosum sub-portions; RT = Reaction-time (milliseconds); Sample size for correlations between memory performance and RT to FA values = 10 subjects for the words experiment and 11 subjects for the pictures experiment;

^a^Level of significance corresponds to a two-tailed test at .05, controlling for age.

* *p*< .05.

**Fig 3:**
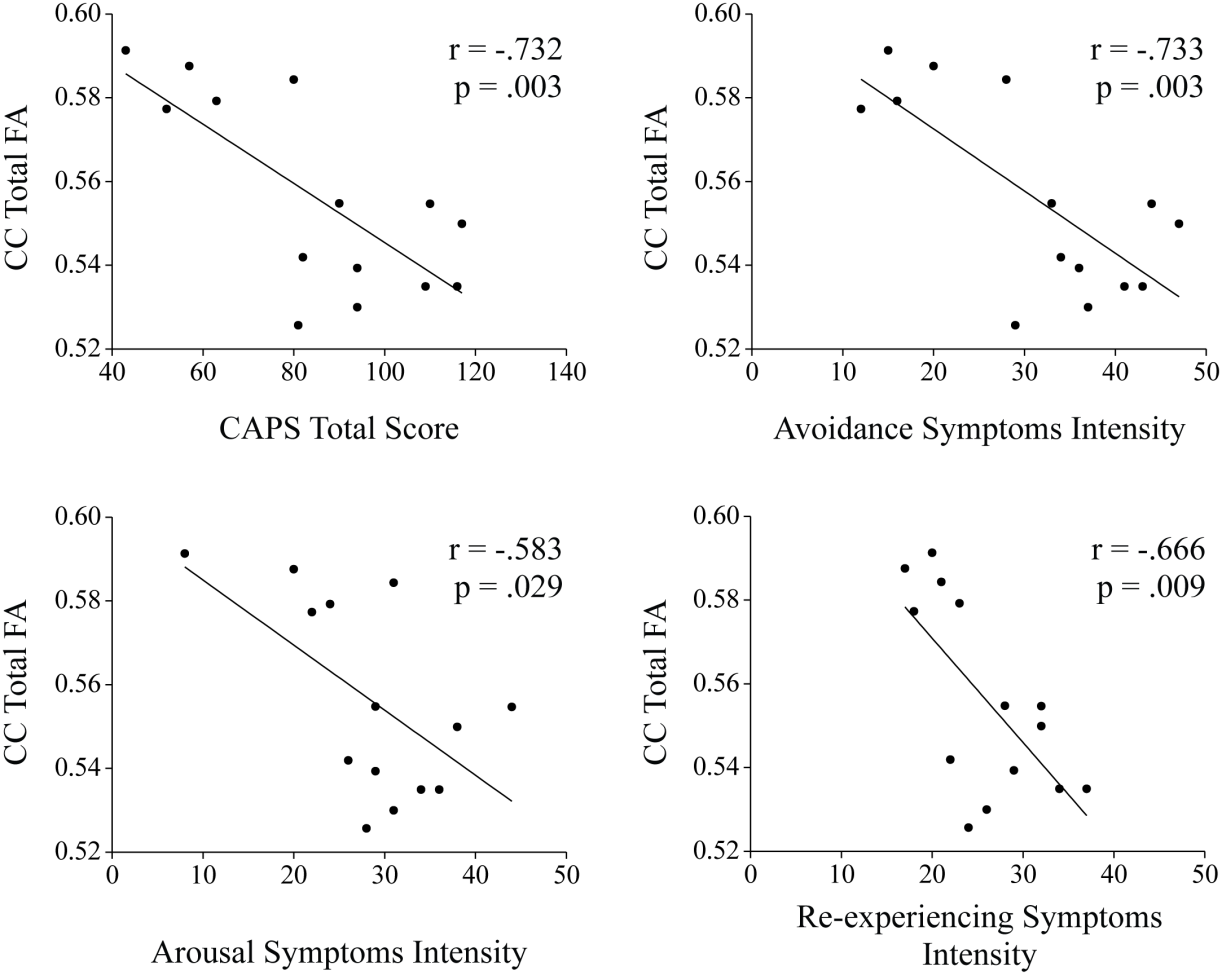
Correlations between symptoms severity and total CC FA values. Significant linear correlations were found between arousal, avoidance and re-experiencing symptoms intensity, as well as to the total CAPS score to total CC FA values.

To answer the question whether imaging and cognitive parameters are associated with PTSD symptom severity we computed the correlation between the total CAPS score and FA of the CC as well as ADI and RT in the PTSD group. As stated before, correlations involving RT in the associative pictorial memory task and the FA values of the anterior CC portions were controlled for age. A significant linear correlation between CAPS symptoms severity (total score) and the total CC FA value was found (r = -.732, p = .003). To test whether this effect is specific to distinctive CC portions, we computed the correlation between the CAPS total score and each CC portion, and found a consistent pattern for most portions (anterior: r = -.559, p = .047, mid-anterior: r = -.669, p = .009, central: r = -.767, p = .001, mid-posterior: r = -.669, p = .009, a trend was found with the posterior portion: r = -.486, p = .092, uncorrected). To test whether this effect is specific to distinctive PTSD symptom clusters (arousal, avoidance and re-experiencing) we computed the correlation between the CAPS sub-clusters and CC FA values using semi-part correlations (results are shown in Fig 3 and Table 4). Notably, correlating a specific symptom cluster (e.g., arousal) with CC FA measures while partialling out the variance from the other two symptom clusters (e.g., avoidance and re-experiencing) resultant in a non-significant correlation for all symptom clusters (see discussion section for interpretation).

**Table 4:** Pearson Correlation Coefficients between Symptoms' severity (CAPS) and Corpus-Callosum Sub-Portion FA Values.

		FA Anterior^a^	FA Mid-Anterior	FA Central	FA Mid-Posterior	FA Posterior^a^	FA Total
PTSD Symptoms	Arousal	-.519	-.511	-.695**	-.672**	-.452	-.583*
	Avoidance	-.506	-.656*	-.722**	-.690**	-.461	-.733**
	Re-experiencing	-.390	-.650*	-.746**	-.529	-.481	-.666**
	Total	-.559*	-.669**	-.767**	-.669**	-.486	-.732**

*Note*. CAPS = Clinician-Administered PTSD Scale; FA = Fractional-anisotropy; CC = Corpus-Callosum; N = 14 for all correlations.

^a^Level of significance corresponds to a two-tailed test at .05, controlling for age.

* *p*< .05,

** *p*< .01.

## Discussion

The results of the current study demonstrated specific correlations between PTSD symptoms severity and behavioral memory measurements (ADI, RT) to white-matter microstructural integrity (FA). The significant correlation between CC FA values and PTSD severity, as measured by the CAPS questionnaire, support the hypothesis that alterations in CC white-matter architecture are associated with core PTSD pathophysiology, rather than a specific symptom cluster. In addition, the findings of the current study significantly expand the results reported in our previous work [9] showing significant correlations between associative pictorial recognition and CC volume, and other studies reporting white-matter alterations in PTSD populations [6–8, 10–11, 24–28].

According to the scheme proposed by Witelson [42] for CC segmentation, compartments of the anterior third, including the rostrum, genu, and rostral body, are assigned to prefrontal, premotor, and supplementary motor cortical areas, respectively. Fibers originating in the motor cortex are assumed to cross the CC through the anterior mid-body, whereas somaesthetic and posterior parietal fiber bundles cross the CC through the posterior mid-body. Since RT in the associative pictorial task was negatively correlated to FA values of the anterior and central portions, it is hypothesized that severe PTSD patients display connectivity alterations in these portions that are translated as a deficit in motor planning, resulting in longer RT. Interestingly, regions with projections through the medial and posterior CC are connected to the prefrontal cortex, and are involved in circuits that mediate the processing of emotional stimuli as well as various memory functions that are known to be impaired in PTSD [25].

Overall, it seems that while the volume of the CC is important for accurate associative recognition of words as well as pictures [9], the anisotropic diffusion (i.e., microstructural integrity) within the white-matter, as tested in the current study, is crucial only for verbal and not for pictorial associative performance. Numerous studies have identified neurocognitive deficits in individuals with PTSD [43–45], most notable are differences in verbal declarative memory, attention, and general intellectual level [46–50]. Impaired verbal processing and memory have been reported in the literature as possible vulnerability factors for PTSD [51]; for example, Bustamante et al. showed that following exposure to a traumatic event, verbal memory performance at baseline appeared not to predict concurrent PTSD severity but did predict later severity of PTSD [52]. Kleim & Ehlers tested for autobiographical memory (which dominantly relies on declarative semantic processing) and reported that people presenting low specificity and over-general memory were at risk to develop PTSD [53]. In this context, the verbal and visual stimuli asymmetry as observed in the current study can be interpreted in several directions. Firstly, there is the possibility of a null finding, or that the variance of pictorial processing in the general population is smaller than the one for verbal memory, thus a larger sample size is needed in order to observe a significant correlation between pictorial memory performance and CC white-matter. Secondly, it is possible that the word associative task is more cognitively demanding (see pictorial superiority effect [54]), thus, requiring a more stable connectivity architecture to allow the recruitment of relevant memory resource allocation regions as compared to the pictorial task. This interpretation suggests that a-more fine-tuned connectivity is required in order to achieve high performance in the associative word recognition task.

Notwithstanding the origin of CC alterations in PTSD is unclear: The question whether anatomical connectivity architecture abnormalities in PTSD patients are a preexisting predisposition due to developmental/genetic factors, or are a consequence of the traumatic exposure itself, remains open. The preexisting predisposition hypothesis relates to premorbid vulnerability, i.e., abnormal white-matter integrity as a risk-factor predisposing individuals to PTSD and predicting its severity. Although the cross-sectional nature of this study prohibits confirmation of this possibility, it is supported by earlier studies of psychiatrically healthy youths with variable family histories of depression and major depressive disorder [55] that demonstrated architectural abnormalities of white-matter in at-risk groups. Given the growing evidence for significant genetic control determining white-matter integrity [56], it is possible that dysfunctional white-matter in PTSD represents a biological marker of genetic risk in this disorder. Future prospective studies are warranted to further explore this hypothesis.

Some general limitations of DTI analysis must be acknowledged. Lateral projections of the CC are difficult to reveal and quantify due to mixture of other fiber bundles with different orientations, especially association fiber bundles that are located in a region lateral to the callosal fibers. This limitation of tractography raises a concern about the validity of the DTI-based fiber reconstruction and resultant parcellation maps. Partial volume effects due to the use of voxel sizes larger than the typical diameter of a specific fiber bundle (or tract) result in apparently reduced diffusion anisotropy and correspondingly lower FA values by averaging white-matter tracts with more isotropic tissues such as gray matter or even CSF. Moreover, there are limitations of the simple diffusion tensor model if fibers merge, branch, or cross. Although the aforementioned challenges emerge as a general concern for fiber tractography, they did not arise as a major obstacle for studying the topology of fiber connections in the human CC. Another limitation to be considered is the small sample size in the current study; Based on the correlation analysis and the sub-group comparisons we concluded that the difference in FA values between extremely-severe and mild-severe PTSD patients is categorical in its nature, yet it is possible that a larger sample size would have revealed a continuous difference rather than a categorical one. Future studies with larger sample size are warranted to further explore the nature of the difference between these groups.

In summary, the current study reveals novel findings regarding the functional relationship between microstructural integrity of specific CC portions and altered cognitive, and specifically memory, performance in PTSD. The correlations observed in the current study have consequences for diagnostic tools and future studies aimed at identifying individuals at risk for PTSD, and in turn, develop individual-based treatments that assist in preventing the chronicity of this disorder.

## Supporting Information

S1 File Complete Data Set used for data analysis in the current study. (ZIP)
